# Long Short-Term Memory Network for Contralateral Knee Angle Estimation During Level-Ground Walking: A Feasibility Study on Able-Bodied Subjects

**DOI:** 10.3390/mi17020157

**Published:** 2026-01-26

**Authors:** Ala’a Al-Rashdan, Hala Amari, Yahia Al-Smadi

**Affiliations:** 1 Biomedical Engineering Department, Faculty of Engineering, Jordan University of Science and Technology, Irbid 22110, Jordan; hkamari18@eng.just.edu.jo; 2 Mechanical Engineering Department, Faculty of Engineering, Jordan University of Science and Technology, Irbid 22110, Jordan; 3 Electromechanical Engineering Technology, Abu Dhabi Polytechnic, Abu Dhabi 20011, United Arab Emirates

**Keywords:** contralateral, IMUs, knee joint angle, LSTM, sensory gadget

## Abstract

Recent reports have revealed that the number of lower limb amputees worldwide has increased as a result of war, accidents, and vascular diseases and that transfemoral amputation accounts for 39% of cases, highlighting the need to develop an improved functional prosthetic knee joint that improves the amputee’s ability to resume activities of daily living. To enable transfemoral prosthesis users to walk on level ground, accurate prediction of the intended knee joint angle is critical for transfemoral prosthesis control. Therefore, the purpose of this research was to develop a technique for estimating knee joint angle utilizing a long short-term memory (LSTM) network and kinematic data collected from inertial measurement units (IMUs). The proposed LSTM network was trained and tested to estimate the contralateral knee angle using data collected from twenty able-bodied subjects using a lab-developed sensory gadget, which included four IMUs. Accordingly, the present work represents a feasibility investigation conducted on able-bodied individuals rather than a clinical validation for amputee gait. This study contributes to the field of bionics by mimicking the natural biomechanical behavior of the human knee joint during gait cycle to improve the control of artificial prosthetic knees. The proposed LSTM model learns the contralateral knee’s motion patterns in able-bodied gait and demonstrates the potential for future application in prosthesis control, although direct generalization to amputee users is outside the scope of this preliminary study. The contralateral LSTM models exhibited a real-time RMSE range of 2.48–2.78° and a correlation coefficient range of 0.9937–0.9991. This study proves the effectiveness of LSTM networks in estimating contralateral knee joint angles and shows their real-time performance and robustness, supporting its feasibility while acknowledging that further testing with amputee participants is required.

## 1. Introduction

Amputees represent a significant percentage of the world’s population, although the specific number of amputees is difficult to ascertain and can only be estimated. Nonetheless, reports reveal that amputation is a common occurrence with a high prevalence [1,2]. For instance, Ziegler-Graham et al. reported a global amputation rate of 1.5 per 1000. As a result, over one million limb amputations are performed annually worldwide—namely, one every 30 s [3]. The lower extremities account for most amputations, and their rate is expected to increase globally in the coming years. While transtibial amputees account for the majority of amputation cases, transfemoral amputees account for a significant proportion worldwide. The primary distinction between TT and TF amputation is the extent to which the knee joint is preserved. Apart from the absence of the knee, transfemoral amputation has a detrimental effect on the strength and muscular balance surrounding the affected hip joint. The severity of hip muscle atrophy is correlated with the residual limb’s length [4,5,6].

Several studies have attempted to estimate lower-limb joint angles using data-driven approaches. Relevant prior work includes the following examples. Previous research on the effective control of patient gait posture emphasized estimating the precise angle of the lower limb’s posture while wearing an orthotic device or prosthesis. For example, Lee and Lee [7] developed a neural network-based posture control technique. The hip and knee angles of the contralateral limb were output by this network, which was trained using the knee angles of the sound leg from volunteer amputees while performing level-ground walking. These gait posture variables were then utilized to construct a regular gait pattern for the prosthetic limb. All steps were developed and simulated offline; the absolute mean errors in prediction were 0.32° and 0.12° for the hip and knee angles, respectively. Lee and Lee [8] also developed a neural network-based gait angle prediction technique; however, the input data consisted of surface electromyography (sEMG) signals from the sound leg. Cascaded neural networks were created in this study; the first network, a radial basis function neural network (RBFNN), used the sEMG signal of the sound leg to predict the knee angle of the same leg, while the second network, a multilayer neural network (MLNN), used the first network’s anticipated knee angle to predict the hip and knee angles of the contralateral leg. The resulting data were then utilized to generate a normal gait pattern. These networks were trained with normal walking data, and the method was developed and validated offline. The calculated gait angles had an absolute average error of 0.25°.

A closer examination of the published research on knee angle estimation reveals that the majority of studies have used machine learning and deep learning techniques to create a continuous knee angle measurement tool, using signals from one leg (e.g., sEMG and kinematic data) to determine the knee angle for the same leg, rather than as a tool to predict the contralateral knee angle [2,9,10,11].

Despite these advancements, previous studies have primarily focused on same-leg joint angle prediction or offline analysis, leaving a significant gap in real-time contralateral knee angle estimation for prosthetic control applications. This study aimed to develop a deep learning model to continuously predict the contralateral knee angle for active prostheses based on kinematic signals from IMUs attached to the sound leg. Long Short-Term Memory (LSTM) networks were selected for this work because they effectively capture long-range temporal dependencies in biomechanical time-series data. Their gated architecture mitigates vanishing-gradient problems seen in standard recurrent neural networks, enabling them to learn full gait cycle dynamics more reliably. These properties make LSTMs particularly suitable for reconstructing joint angle trajectories from IMU signals.

Because all training and evaluation in this work were performed on able-bodied participants, the study is presented as a feasibility investigation rather than a clinical validation of amputee gait. The experimental results revealed that the predicted knee angle signal could be used to drive the lower limb prosthesis, resulting in a real-time, reasonable, and robust knee angle estimation algorithm. However, this early demonstration reflects functional feasibility in able-bodied gait rather than a fully validated prosthesis control interface.

In this work, the model is presented as a feasibility demonstration for future integration into active prosthesis control. The real-time tests conducted in LabVIEW 2017 were intended to validate functional responsiveness rather than to serve as a finalized control interface.

This study was approved by the Institutional Review Board (IRB) of Jordan University of Science and Technology and King Abdullah University Hospital (Ref. 20/137/2021, approval date: 2 March 2021). All procedures complied with the Declaration of Helsinki. Written informed consent was obtained from all participants prior to data collection, and participation was voluntary. Data confidentiality was maintained according to institutional and ethical regulations.

## 2. Materials and Methods

**Figure 1:**
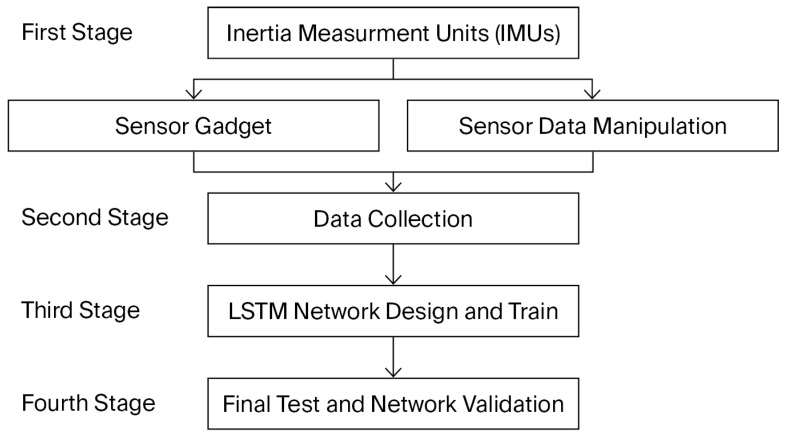
The flowchart of the present research.

All experimental procedures described in this section were conducted exclusively on able-bodied participants, as the present work represents a feasibility investigation rather than a clinical validation study. The flowchart of this work is shown in Figure 1. Each of the phases will be explained in detail in the following sections.

### 2.1. Sensor Device and Data Manipulation

The experimental procedure was based on data collected from inertial measurement units (IMUs). IMUs are wearable, portable, lightweight, and safe and can be used at home since they are inexpensive and simple to handle. IMUs measure acceleration and angular rates in their own three-dimensional local coordinate frames [12]. The MPU6050 (by InvenSense, San Jose, CA, USA) was used. This is a 6-axis device with a 3-axis accelerometer and 3-axis gyroscope.

**Figure 2:**
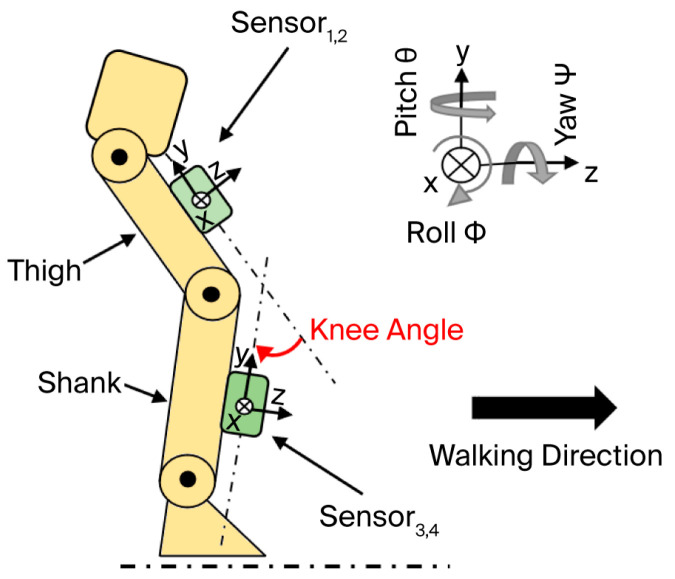
Sensor locations and orientation during level-ground walking; the figure is adapted from [13].

Four MPU6050 sensors were used to collect data from both legs simultaneously; this required a suitable device in which the sensors were attached to the thigh and shank center of masses (COMs) of each subject. Figure 2 shows the sensor’s orientation during level-ground walking.

The center of mass (COM) of the thigh and shank segments was approximated using anthropometric ratios described [14], where the thigh COM is located at 43% of segment length from the proximal end, and the shank COM at 38% from the proximal end. These standardized ratios are widely used in gait biomechanics and enable reproducible placement across subjects.

The sensors were linked through I2C pins on a National Instruments myRIO-1900 (National Instruments Corporation, Austin, TX, USA), which collected data at a sampling rate of 200 Hz; the data were manipulated using LabVIEW 2017. The acceleration and angular velocity data obtained from the sensor are noisy; the acceleration data consist of a three-dimensional combination of linear acceleration and gravitational acceleration; and the acceleration and angular velocity data are fused to determine the segment angles. Next, the raw data obtained from the MPU6050 sensor were processed and manipulated.

The MPU6050, as with other sensors, is calibrated before use by eliminating the zero error, which occurs when the sensor reports a miniscule angle even if it is perfectly level. This error is eliminated by implementing an offset to the raw accelerometer and gyroscope sensor data. The offset is adjusted until the triaxial gyroscope readings reach zero, and the accelerometer records gravity acceleration pointing straight downwards. After this, the data are saved in the LabVIEW 2017 [15,16].

The noise generated by IMUs during sensor movement is a significant problem. Another source of sensor signal interference is vibration caused by ground contact at the onset of the stance phase, i.e., heel strike. As a result, the calibration step was immediately followed by a filtering stage. Gravity compensation and segment orientation estimation were performed using a quaternion-based orientation filter following [17]. This algorithm fuses tri-axial accelerometer and gyroscope measurements to compute a drift-reduced 3D orientation estimate of each IMU. The gravitational component was then removed from the accelerometer signals using the estimated quaternion, allowing reconstruction of linear acceleration and segment angles in the global reference frame. In the literature [18,19], a Butterworth low-pass filter is recommended. In our work, the filter order and cutoff frequency were determined to be the second order and 4 Hz, respectively. The filter was built in the LabVIEW program with a point-by-point operation necessary for real-time signal processing.

Another issue is the acceleration measured by IMUs, since they measure local acceleration rather than global acceleration. Thus, gravity compensation is necessary to calculate the linear acceleration in the global reference system. In this work, the compensation method applied is the quaternion base correction described in [17].

Since the kinematics of the knee are characterized by a large sagittal range of motion and a limited range in the coronal and transverse planes, the sagittal plane knee angle was calculated using the MPU6050 [20].

Although human walking includes minor out-of-plane motions, sagittal knee angle was isolated using a quaternion-based orientation filter [18]. The IMU orientation was estimated in 3D, then re-projected onto the sagittal plane using the segment coordinate system. The knee angle was computed as the relative rotation between the thigh and shank about the flexion–extension axis after removing abduction–adduction and internal–external rotation components.

The MPU6050 module is not a tilt angle measurement device; thus, the sensor’s raw data must be utilized in a formula to determine the tilt angle [12]. The knee angle is defined as the difference between the angles of the thigh and shank segments [20]. For each trial, the triaxial linear acceleration, triaxial angular velocity, thigh angle, shank angle, and knee angle were recorded in an Excel sheet.

(1) $$\theta_{Knee}=\theta_{Thigh}-\theta_{Shank}$$

### 2.2. Data Collection and Experimental Setup

Twenty able-bodied subjects (10 males and 10 females with an age of 26 ± 6 years, weight of 70 ± 16 kg, and height of 170 ± 9 cm) participated in this study, enabling the collection of reference gait kinematic data for the training of the proposed network. None of the subjects had experienced lower limb amputation or impairments, and they did not have severe gait disorders that could affect their walking abilities or gait patterns. Moreover, they did not have significant noncorrected vision impairments, hip dysplasia, diabetes, heart problems, spinal cord diseases, back pain, or lower extremity congenital abnormalities. No pregnant women were permitted to participate.

**Figure 3:**
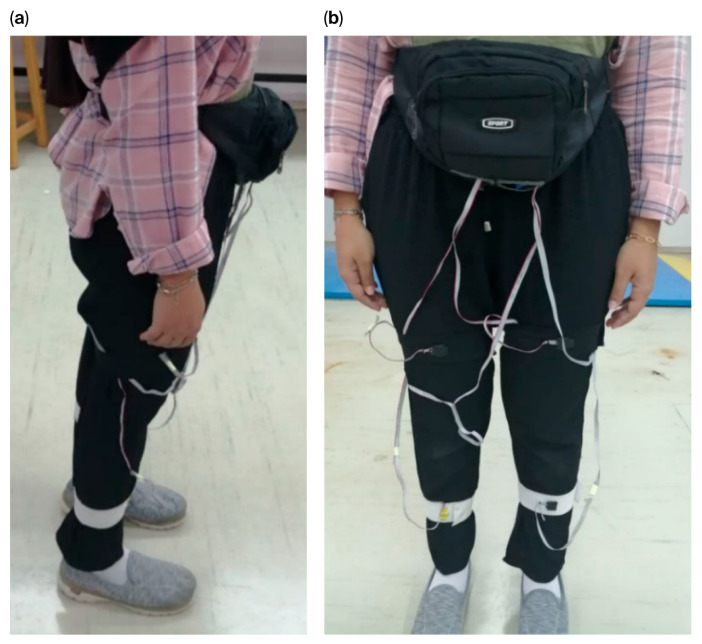
Sensor device attached while performing level-ground walking: (**a**) side view, (**b**) front view.

The participants wore the sensor device during the experimental session, as shown in Figure 3. The sensors were attached to the anterior portion of the volunteer’s shank and the thigh COM, and they were then instructed to perform a ground-level walking activity at a self-selected speed on a 12 m path. Additionally, a 30 s rest period was included between each session. Each participant was subjected to 15 repeated sessions. The triaxial angular velocity and linear acceleration of the thigh and shank segments, as well as the shank, thigh, and knee angles for both legs, were collected as reference gait kinematic data. These were subsequently transferred to the MATLAB 2021 software for use in the training of the LSTM network.

Hyperparameters were tuned empirically using grid search across the ranges (hidden units: 50–200; dropout: 0.1–0.5; learning rate: 1 × 10^−4^–1 × 10^−2^; batch size: 20–100). The selected configuration (150 neurons, dropout 0.3, learning rate 0.001, batch size 50) achieved the lowest validation loss without overfitting. Increasing neurons beyond 150 caused overfitting, while lower values reduced prediction accuracy.

### 2.3. LSTM Network Design and Training

The long short-term memory (LSTM) network is a type of RNN that was developed in 1997 by Hochreiter and Schmidhu [21], designed to learn long-term dependencies. It consists of a feedback link and gated memory cells that provide steady gradients or error flow; this resolves the issues of vanishing and explosion, thus enabling the network to learn temporal relationships covering over a long period of time steps. The hidden layer is trained using an architecture consisting of input, forget, and output gates. LSTM cells exchange data and categorize them into two groups; one group is kept while the other is forgotten, depending on their significance. Therefore, LSTM is a unique method of storing and retrieving memory across cells/neurons.

**Figure 4:**
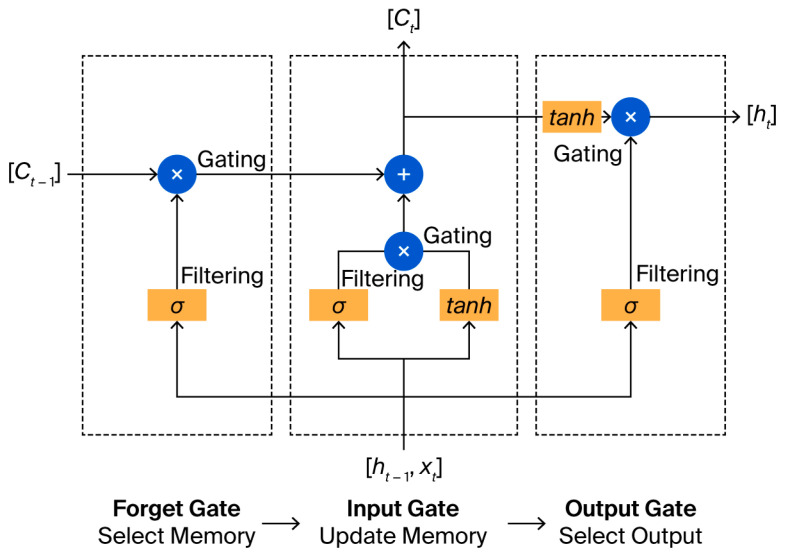
Standard structure of an LSTM cell [23].

LSTM differs from a traditional RNN in that it retains the hidden state data, combines them with the current data to determine outcomes, and updates the cell states, in addition to integrating the prior output and the current input. This implies that the RNN’s current output is determined by the present input and the prior output, while the LSTM’s current output is determined by the current input, prior output, and prior state. The RNN cell will provide the hidden value, while the LSTM cell will return the hidden value together with new cell states. LSTM networks consist of many connected LSTM cells, and each of them is composed of three essential gates. These gates determine whether the information from the past/present flows through. The incoming data within each cell pass through three stages via the three different gates [22]. The structure of the LSTM neural network is shown in Figure 4.

#### 2.3.1. Network Design

The LSTM network structure was adjusted experimentally and determined to be consistent across all prediction types. Hyperparameters were tuned empirically using grid search across the ranges (hidden units: 50–200; dropout: 0.1–0.5; learning rate: 1 ×10^−4^–1 × 10^−2^; batch size: 20–100). The selected configuration (150 neurons, dropout 0.3, learning rate 0.001, batch size 50) achieved the lowest validation loss without overfitting. Increasing neurons beyond 150 caused overfitting, while lower values reduced prediction accuracy. The input for each network is listed in Table 1.

The choice of LSTM depth, number of hidden units, and sequence window length was guided by established practices in sequential human-motion prediction. A two-layer LSTM structure was selected to provide sufficient representational capacity without introducing unnecessary complexity, while the 150 hidden units were chosen to balance model expressiveness with computational efficiency. The sequence window length was set to capture a meaningful portion of the gait cycle, enabling the model to learn both short-term and long-term temporal dependencies inherent in IMU kinematic data. These design choices align with prior work in gait angle estimation and biomechanical time-series modeling.

The 15-dimensional input vector consisted of 12 raw IMU signals (triaxial acceleration and triaxial angular velocity from the thigh and shank sensors) and 3 processed kinematic angles (thigh angle, shank angle, and knee angle). These three angles were computed from the IMU measurements using the filtering and fusion procedures described in Section 2.1.

#### 2.3.2. Network Training

The Adam optimization technique was employed to train the network, and a stop loss constraint was applied to the training process by assessing the validation loss throughout the validation stages. If the validation loss did not improve over the previous ten validation checks, the training was terminated.

The network was trained using fifty steps from each volunteer, with the input data divided as follows: 80% were used for training, and 20% were used for testing. The networks were stored and used in the real-time testing stage. The high-performance computing system used to train the networks consisted of an Intel^®^ Xeon^®^ Gold 6226R processor with a processing speed of 2.9 GHz, 16 cores, and 64 GB installed RAM, located in the Autonomous Platforms lab at Jordan University for Science and Technology.

#### 2.3.3. Network Evaluation

In order to evaluate each network’s performance, the RMSE was used, as well as the Pearson correlation coefficients for the training and validation datasets. The networks were evaluated using unseen subjects. The test set consisted solely of the two individuals not included in training or validation. For each subject, four walking trials of identical length and sampling frequency were collected. No overlapping sequences with the training data were used.

### 2.4. Real-Time Testing

A real-time test using the constructed networks was conducted as a final assessment. In the LabVIEW software, the method was implemented in real time with the original sensor system. The weight and bias values obtained upon training the LSTM networks were saved, and the LSTM algorithm was used to analyze the IMU data received in real time during walking to predict the knee angle. A real-time test using the constructed networks was conducted as a final assessment.

In this study, ‘real-time’ refers to the continuous online generation of knee angle predictions from the incoming IMU data stream on a sample-by-sample basis, without buffering or offline computation. During the walking trials, the system produced predictions simultaneously with data acquisition, and no perceptible delay was observed in the displayed output. Because the aim of this work is to demonstrate feasibility, a detailed latency measurement was not included; such characterization will be carried out in future work when the algorithm is implemented on embedded prosthesis hardware.

A limitation of this study is the small number of subjects (*n* = 2) included in the real-time testing phase. Although results were consistent, future work will include a larger cohort to further validate the model’s performance across a broader range of users.

Moreover, the present study is limited to steady level-ground walking. Future work will include variable-speed walking, incline negotiation, directional changes, and obstacle conditions to ensure robustness for real-world prosthetic applications.

As such, the real-time evaluation presented here demonstrates functional feasibility in able-bodied users and does not represent a validated prosthesis control implementation.

## 3. Results and Discussion

Two LSTM networks were trained and tested to estimate the contralateral leg’s knee angle; one, referred to as LR, utilized left leg kinematic data to predict the right leg knee angle, while the other, referred to as RL, utilized right leg kinematic data to predict the left leg knee angle. The estimated angles were obtained at the same time instance as in the kinematic data.

### 3.1. Training and Testing Results

During the training phase of the LSTM networks, the RMSE loss function was employed. The stop loss constraint was applied to the training process by assessing the validation loss throughout the validation stages; the training was terminated if the validation loss did not improve over the previous ten validation checks.

**Figure 5:**
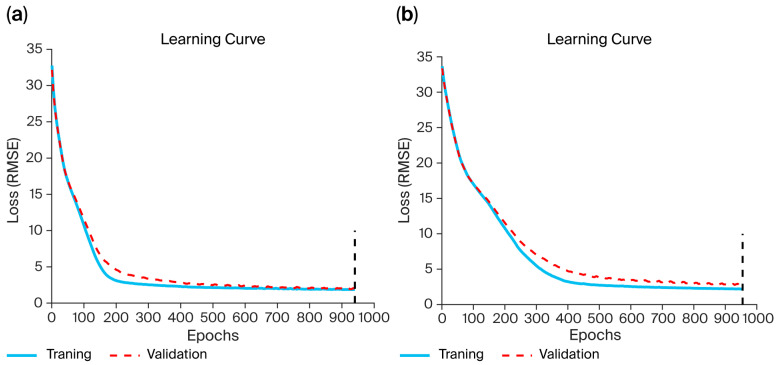
Learning curves of (**a**) RL LSTM network and (**b**) LR LSTM network.

Figure 5 illustrates the learning curves, demonstrating the performance of the RL and LR LSTM networks throughout the training period. The *y*-axis represents the loss value, which indicates how well the model performs after each epoch, and the *x*-axis represents the epoch number. The blue line represents the training loss, the red dashed line represents the validation loss, and the vertical dashed black line represents the maximum number of epochs. The two curves are stable, smooth, and systematically decreasing. In addition, there is no overfitting between the training and validation data, since the training and validation RMSEs are very close.

Table 2 displays the evaluation parameters for the training and validation datasets, the training durations, and the maximum numbers of training epochs for the LR and RL networks. The difference in training time between the two networks was four minutes; this was deemed small compared to the overall training duration of each network, which was more than one hour. This time difference was proportional to the maximum number of epochs for each network.

**Figure 6:**
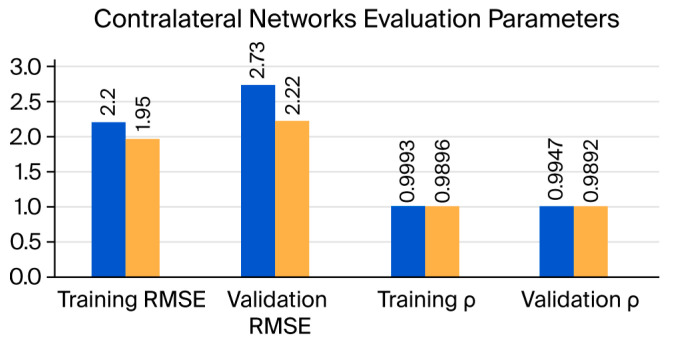
Comparison of evaluation parameters for two contralateral networks.

Figure 6 presents the temporal comparison between actual and predicted knee angles, offering visual insight that complement the numerical RMSE values in Table 2. It also illustrates the variations in the evaluation parameters as bar charts, including the correlation coefficient (ρ) and the RMSE between the actual knee angle and the estimated knee angle obtained by each network. The evaluation parameters for the training dataset are slightly larger than those for the validation dataset, which is typically observed for deep learning networks. The correlation coefficients (ρ) for the LR and RL networks are 0.9892 and 0.9947, respectively. As both coefficients are ≥0.8, this indicates a strong correlation between the actual and predicted knee angles in both networks. The RMSE is 2.73° and 2.22° for the LR and RL networks, respectively. The errors for the RL network are somewhat smaller than those for the LR network by about 0.5°, which is a reasonable difference.

It is important to note that all training and validation were performed on able-bodied subjects; therefore, these performance comparisons reflect feasibility in non-amputee gait rather than validated performance in amputee gait.

**Figure 7:**
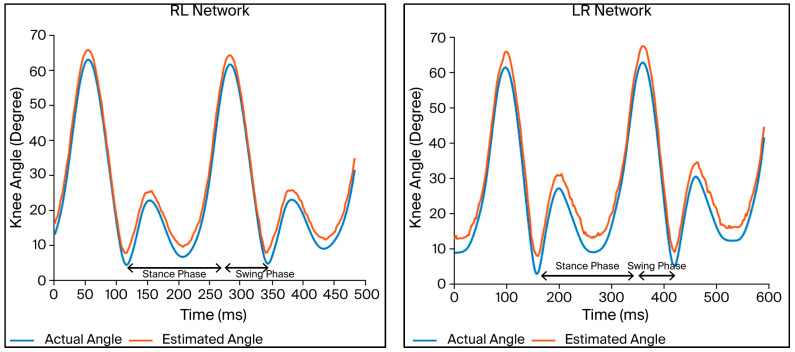
Comparison of actual angles and estimated angles obtained using contralateral networks for segment of validation data.

Figure 7 compares the knee joint angles in terms of the actual data and validation data for the two LSTM networks. The curves in each plot exhibit the same trends. The estimated knee joint angles are highly similar to those obtained using the sensor system, indicating that the peak in flexion occurs at the middle of the stance phase and in the second half of the swing phase. The findings indicate that the knee joint angles are correctly estimated, since they are comparable to normal knee angle patterns seen while walking.

It is important to note that the comparison between the LR and RL networks is descriptive, as the study design did not include repeated walking trials that would enable statistical hypothesis testing. The observed difference in RMSE between the two models therefore reflects model behavior on available trials rather than a statistically validated performance advantage. Future work will incorporate repeated measurements across multiple sessions to support meaningful statistical comparison.

### 3.2. Real-Time Testing Results

**Figure 8:**
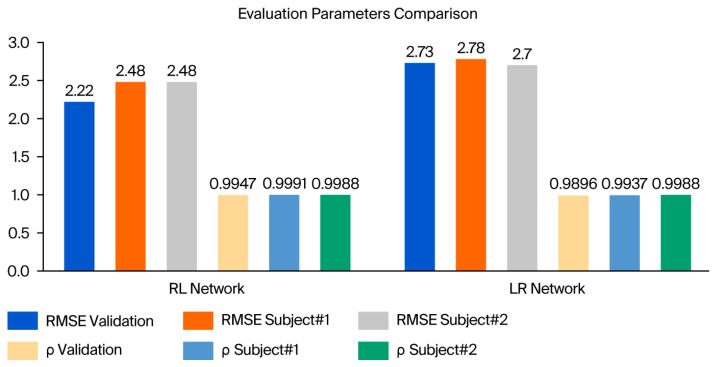
Comparison of real-time evaluation parameters with validation parameters for RL and LR LSTM networks.

The results of the real-time tests of the LR and RL LSTM networks, covering four individual trials, are listed in Table 3 and Table 4, while Figure 8 illustrates the model’s real-time tracking behavior across the gait cycle, enabling visualization of the temporal prediction performance, it also provides a comparison between the validation and real-time evaluation parameters in a bar chart.

For the LR LSTM network, the average RMSEs were 2.78° and 2.70° for subject 1 and subject 2, respectively. The correlation coefficients (ρ) were 0.9937 and 0.9988 for the two subjects, respectively. Meanwhile, for the RL LSTM network, the average RMSEs were 2.48° and 2.48° for subject 1 and subject 2, respectively. The correlation coefficients (ρ) were 0.9991 and 0.9988 for the two subjects, respectively. Upon comparing these findings to the validation values of both networks—i.e., an RMSE of 2.73°, 2.22° and ρ of 0.9896, 0.9993 for the LR and RL networks, respectively—it is clear that both the RMSE and ρ are within the same ranges, but the RMSE is slightly higher. The reason for this increase in the RMSE is that the algorithm’s hidden parameters (weighted and basis) were chosen during the training phase to improve the overall validation set performance, whereas the real-time test set contained data from subjects not seen in either the training or validation stages.

Unlike prior work that estimates knee angle of the same leg using sEMG or IMU signals [7,8,9,10,11], the present study predicts the contralateral knee angle in real time. This is highly relevant to prosthetic knee control, where the sound leg governs the motion of the artificial knee. Existing models reporting lower RMSE values generally perform offline estimation or rely on electromyography, which is not practical for real-time prosthesis control. The proposed approach achieves competitive accuracy using IMUs alone and is directly deployable in embedded prosthetic systems.

**Figure 9:**
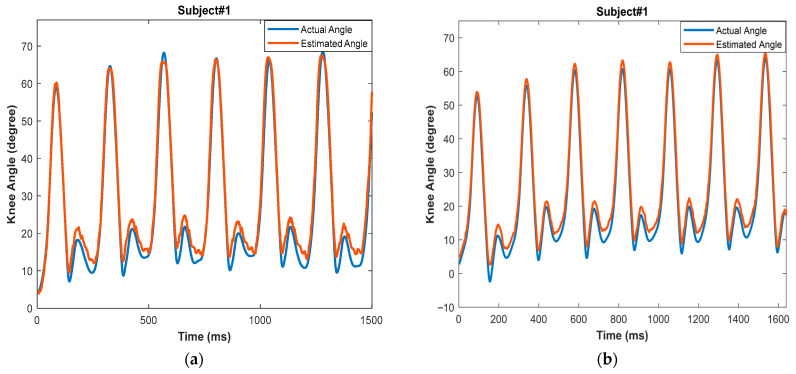
Real-time test results for (**a**) LR LSTM network and (**b**) RL LSTM network.

Figure 9 provides a comparison of the knee joint angles from the actual data and the real-time test data from trial #1 for subject #1 across the two networks. As in the validation dataset, the knee angles are correctly estimated using the developed system, since they are comparable to normal knee angle patterns seen while walking.

The predicted knee angles exhibit a small positive bias relative to the reference signals. This behavior is consistent with known effects in IMU-based angle estimation, where slight orientation misalignments, segment calibration errors, and small integration drifts can shift the reconstructed joint angles upward. Additionally, minor timing offsets between the IMU-derived angles and the reference signal may contribute to this systematic difference.

A visual inspection of the predicted and actual knee angle curves (i.e., Figure 7 and Figure 9) revealed that the largest errors tended to occur during rapid transitions in the gait cycle, particularly around the flexion peak and the subsequent extension phase. These regions involve faster angular changes, which are generally more challenging for recurrent models to track. In contrast, periods of relatively steady motion, such as mid-stance, showed lower prediction errors. Although the dataset did not include labeled gait events for detailed phase-specific analysis, these qualitative observations align with known patterns of error concentration during high-dynamics gait segments.

## 4. Conclusions

Recently, an increase in the number of lower limb amputees has been observed globally, and transfemoral amputation is the second most common type of lower limb amputation. Therefore, an improved prosthetic knee joint is required, which could improve the amputee’s ability to resume activities of daily living. This research proposes LSTM models for the effective estimation of the knee angle during level-ground walking, utilizing the kinematic data of the intact leg to predict the motion pattern (knee angle) of the affected one. Because the current dataset consists exclusively of able-bodied individuals, the present work should be interpreted as a feasibility investigation rather than a clinically validated prosthesis control solution. The predicted knee angle provides the required information for the real-time control of active transfemoral leg prostheses.

A sensory apparatus comprising four IMU sensors was developed in order to collect the required kinematic data to train the LSTM network; these data were then input to the generated model in a real-time application. The kinematic data were subsequently filtered and manipulated in order to obtain the thigh segment angle, shank segment angle, and knee angle.

Contralateral LSTM models were created using data from one leg to estimate the knee angle of the opposite leg at the same time point; the results indicate a small difference in predicted error between the LR and RL models, amounting to about 0.5°. The results of the real-time tests reveal a small increase in the root mean square error (RMSE) as compared to the validation error, which is due to the introduction of new data into the created model, as opposed to using the same data as in the training and validation process.

Quantitatively, the proposed LSTM networks achieved RMSE values of 2.73° for the LR model and 2.22° for the RL model, with correlation coefficients exceeding 0.94 across all test subjects. These numerical outcomes confirm that the predicted knee angle trajectories remain closely aligned with the reference measurements and demonstrate sufficient accuracy to support future real-time prosthetic control implementations.

This study has several limitations. The real-time evaluation was conducted using only two able-bodied participants, and the system has not yet been tested with transfemoral amputees, who may exhibit different gait dynamics and sensor characteristics. Additionally, the real-time testing was performed in a controlled laboratory environment using a LabVIEW-based interface rather than an embedded prosthesis controller. These factors indicate that the current work should be considered a feasibility demonstration, and future studies are required to validate the approach on a larger and more diverse population and within a fully integrated prosthetic control system.

To summarize, this study proves the potential of LSTM networks to continuously estimate the contralateral knee joint angle with a low root mean square error and a high correlation coefficient in able-bodied subjects. The real-time tests reveal the model’s reasonability and robustness, under controlled laboratory conditions. Since the purpose of estimating the knee angle is to control the artificial knee in a transfemoral prosthesis, future attempts may include further design modifications and interactions with controllers, as well as testing the models’ performance during integration with a real-time control system. This could provide valuable reference signals for active knee joint actuators and motivate further research in this field.

## Figures and Tables

**Table 1:** LSTM network types with the network’s inputs and outputs.

Network Type	Network Input									Network Output	
	R-Sh Acc + Av (Triaxial)	R-Th Acc + Av (Triaxial)	L-Sh Acc + Av (Triaxial)	L-Th Acc + Av (Triaxial)	R Sh + Th Angle	L Sh + Th Angle	R-Knee Angle	L-Knee Angle	Total Input #	R-Knee Angle	L-Knee Angle
Contralateral RL	×	×			×		×		15		×
Contralateral LR			×	×		×		×	15	×	

**Table 2:** Evaluation parameters, training times, and epoch numbers for contralateral networks.

Network Type	Training Time (Hour)	Number of Epochs	Training RMSE	Validation RMSE	Training ρ	Validation ρ
LR Network	1:08:38	955	2.20	2.73	0.9896	0.9892
RL Network	1:04:46	940	1.95	2.22	0.9993	0.9947

**Table 3:** Real-time evaluation of LR LSTM network for two test subjects.

Trial	Subject 1		Subject 2	
	RMSE	ρ	RMSE	ρ
Trial 1	2.85	0.9955	2.74	0.9963
Trial 2	2.59	0.9933	2.67	0.9937
Trial 3	2.92	0.9908	2.93	0.9952
Trial 4	2.75	0.9952	2.46	0.9935
Average	2.78	0.9937	2.70	0.9937

**Table 4:** Real-time evaluation of RL LSTM network for two test subjects.

Trial	Subject 1		Subject 2	
	RMSE	ρ	RMSE	ρ
Trial 1	2.57	0.9994	2.48	0.9991
Trial 2	2.46	0.9989	2.61	0.9985
Trial 3	2.50	0.9990	2.54	0.9984
Trial 4	2.40	0.9992	2.29	0.9993
Average	2.48	0.9991	2.48	0.9988

## Data Availability

The data that support the findings of this study are available from the corresponding author upon reasonable request.
